# First report of the genus *Pelecystola* Meyrick (Lepidoptera, Tineidae) in China, with description of a new species

**DOI:** 10.3897/zookeys.1046.68329

**Published:** 2021-06-22

**Authors:** Lin-Lin Yang, Hou-Hun Li

**Affiliations:** 1 Institute of Plant Protection, Henan Academy of Agricultural Sciences, Henan Key Laboratory of Crop Pest Control, Key Laboratory of Integrated Pest Management on Crops in Southern Region of North China, Zhengzhou 450002, China College of Life Sciences, Nankai University Tianjin China; 2 College of Life Sciences, Nankai University, Tianjin 300071, China Institute of Plant Protection, Henan Academy of Agricultural Sciences Zhengzhou China

**Keywords:** CAD, COI, morphology, molecular phylogeny, new record, taxonomy, wingless

## Abstract

The genus *Pelecystola* Meyrick, 1920 and the species *Pelecystola strigosa* (Moore, 1888) are newly recorded from China, and *Pelecystola peculiaris***sp. nov.** is described as new to science. Adults, head, venation, and genitalia of the two species are illustrated. A molecular phylogenetic analysis is presented to ascertain the generic affiliation of the new species. Forty-four species of 38 genera in Tineidae are analyzed using maximum likelihood methods based on one mitochondrial (COI) and two nuclear gene fragments (CAD and wingless). DNA barcodes of the two species are provided, and the genetic distance of barcode divergence among four species of *Pelecystola* is calculated.

## Introduction

The genus *Pelecystola* was erected by Meyrick (1920) with *Pelecystola decorata* Meyrick, 1920 as its type species. It is represented by seven species, distributed in Africa, Asia, Europe, and North America (Gozmány and Vari 1973; Sakai 2002, 2013; Davis and Davis 2009; Lindeborg and Bengtsson 2009; Robinson 2009; Gaedike and Tokár 2010). *Pelecystola* is characterized by the deeply bilobed uncus, the valva being divided into cucullar and saccular lobes, and usually possessing an elongate stalk which enlarges apically to form a pedunculate pectinifer in the male genitalia, and the corpus bursae having a V-shaped signum consisting of a pair of slender arms in the female genitalia. It has not been assigned to any subfamily, although it is sometimes referred to the Scardiinae (Gozmány and Vári 1973; Sakai 2002). Herein, the genus *Pelecystola* is reported from China for the first time, and *Pelecystola peculiaris* sp. nov. is described as new to science. A molecular phylogenetic analysis employing 44 tineid species as ingroups is presented to ascertain generic affiliation of the new species.

## Materials and methods

### Taxon sampling

The specimens examined were collected using 250-W high-pressure mercury lamps on white sheet. Samples for DNA extraction were collected during the last two years. DNA barcodes of four specimens of *Pelecystola peculiaris* sp. nov. and five specimens of *P. strigosa* (Moore, 1888) were sequenced to calculate the minimum genetic *p*-distance barcode divergence. Three gene fragments (COI: cytochrome c oxidase subunit I, CAD: carbamoyl phosphate synthetase domain protein, and wingless) of five species (*Pelecystola peculiaris* sp. nov., *P. strigosa*, *Tinissa indica* Robinson, 1976, *Micrerethista denticulata* Davis, 1998, and *Opogona* sp.) were sequenced. The specimen data of the sequenced species and GenBank accession numbers are listed in Suppl. material 1: Table S1. To reconstruct a molecular phylogenetic tree of Tineidae, 38 genera and 44 species were selected as ingroups, and two species, *Tischeria ekebladella* Bjerkander, 1795 in Tischeriidae and *Azaleodes micronipha* Turner, 1923 in Palaephatidae were chosen as outgroups. Among these ingroups, 34 species in 30 genera are attributed to 12 subfamilies, 10 species in eight genera are unplaced to subfamily follow classifications of recent reviews (Robinson 2009; Mutanen et al. 2010; van Nieukerken et al. 2011; Regier et al. 2015). The species used in the phylogenetic analysis are listed in Suppl. material 2: Table S2.

The type specimens are deposited in the Insect Collection, College of Life Sciences, Nankai University (**NKU**), Tianjin. The voucher specimens of the sequenced species are preserved in the Insect Collection, Institution of Plant Protection, Henan Academy of Agricultural Sciences (**HAASM**), Zhengzhou, China.

### Morphological analyses

Morphological terminology in the descriptions follows Davis and Davis (2009). Genitalia dissection and mounting methods follow Li (2002), and head dissections were carried out following the methods described by Lee and Brown (2006). Photographs of the adults were taken with a Leica M205A stereomicroscope, and photographs of genitalia were taken with a Leica DM750 microscope plus Leica Application Suite 4.6 software. All photographs were refined with Photoshop CS4 software.

### DNA extraction, PCR amplification, and sequencing

DNA was extracted from dried and alcohol-preserved specimens with the head, genitalia, and wings mounted on slides as vouchers. Total genomic DNA of the specimens was extracted using Qiagen DNeasy Blood & Tissue Kit. DNA amplifications of selected genes were carried out using the primers as listed in Suppl. material 3: Table S3. PCR was performed in 25 μl reaction volume (Suppl. material 4: Table S4), and the PCR conditions were presented in Suppl. material 5: Table S5. PCR products were electrophoresed on 1% agarose gel. Products that were in accordance with the expected length were then sent to Sangon Biotech (Zhengzhou, China) for sequencing.

### Phylogenetic analyses

The sequences were assembled using DNAMAN v. 8 (Copyright 2018 Lynnon Biosoft) and deposited in GenBank and BOLD systems. Multiple sequence alignments were performed with BioEdit v. 7.2.5 (Hall 1999). The quantification of sequence divergences was conducted using the Kimura two-parameter model (K2P) method in MEGA X (Kimura et al. 2018). Data set concatenation, and best evolutionary model selection and phylogenetic inference were processed using PhyloSuite v. 1.2.2 (Zhang et al. 2020): best partitioning scheme and evolutionary models for nine pre-defined partitions were selected using PartitionFinder2 (Lanfear et al. 2017), with the greedy algorithm and AICc criterion; maximum likelihood (ML) phylogenetic analyses for the concatenated gene data set was inferred in IQ-TREE (Nguyen et al. 2015) under Edge-unlinked partition model for 5000 standard bootstraps, as well as the Shimodaira–Hasegawa-like approximate likelihood-ratio test (Guindon et al. 2010).

## Results

**Figure 1.**
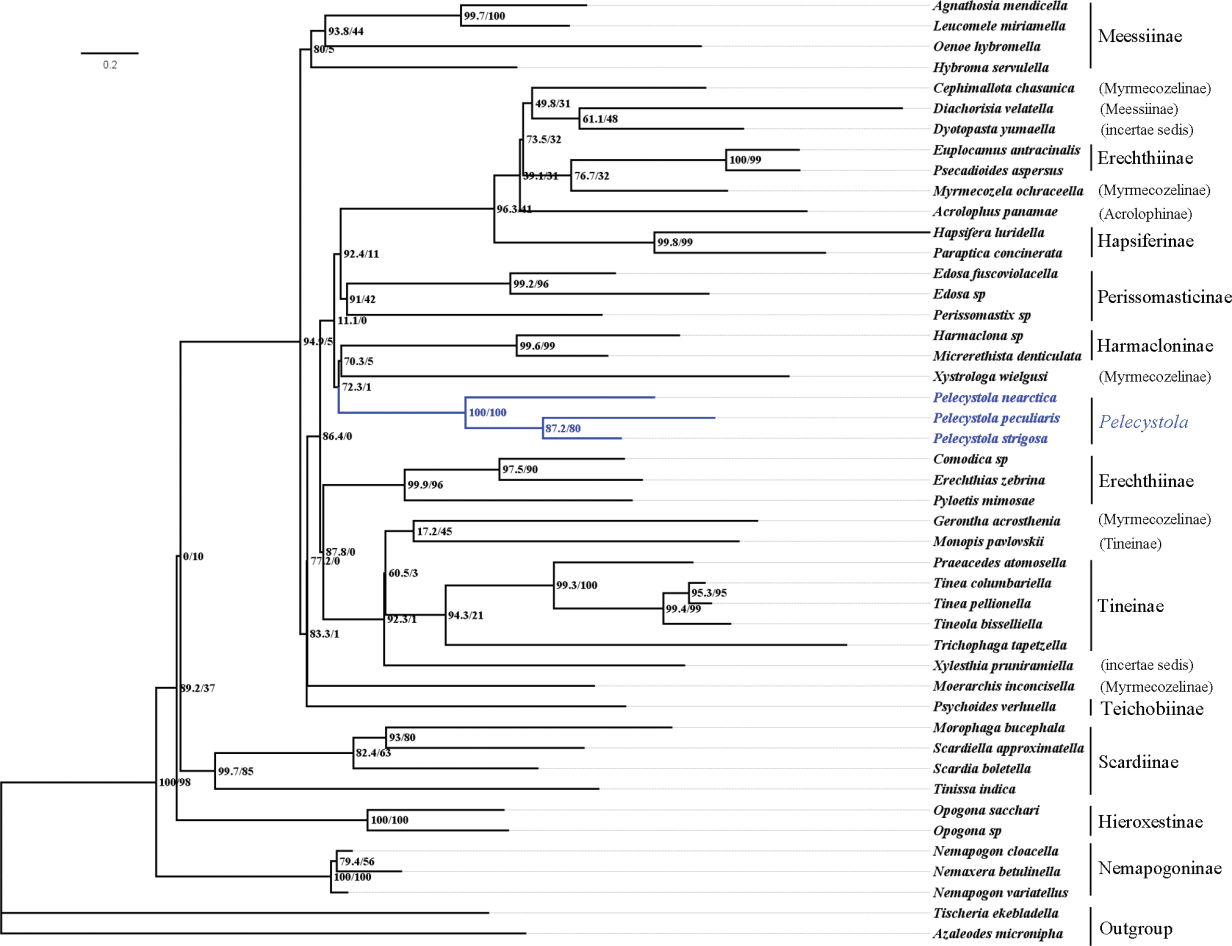
Maximum Likelihood (ML) tree of Tineidae reconstructed by IQ-TREE, based on the concatenated DNA data set (COI, CAD, wingless). Numbers on the nodes refer to the posterior probability (PP) values (%) on the left and bootstrap (BS) values (%) on the right.

The phylogenetic analysis recovered 46 sequences of COI, CAD, and wingless genes. After alignment and the deletion of ambiguous sites, COI had a size range of 657 bp, CAD of 849 bp, and wingless of 402 bp. The results are summarized in the maximum likelihood tree (Fig. 1). It strongly corroborates that the new species belongs to the genus *Pelecystola*, as *P. peculiaris* sp. nov., *P. strigosa*, and *P. nearctica* Davis & Davis, 2009 group together as a strongly supported clade (SH-aLRT = 100%, bootstrap support = 100%). However, the subfamilial affinity of *Pelecystola* remains unresolved.

The genetic distance barcode divergence among four species (*Pelecystola peculiaris* sp. nov., *P. strigosa*, *P. nearctica*, and *P. fraudulentella* (Zeller, 1852) was based on the pairwise analysis of 17 sequences. Sequence divergences among individuals (Table 1) indicated that minimal interspecific distances ranged from 9.39 to 11.62%, and the maximal intraspecific distances ranged from 1.00 to 5.58%.

**Table 1.** Percentage of divergence in the cytochrome c oxidase subunit I (COI) gene sequences of the *Pelecystola* species.

	1	2	3	4
1 *Pelecystola peculiaris* sp. nov.	**0.00–1.51**			
2 *P. strigosa*	11.62–14.00	**0.50–5.58**		
3 *P. nearctica*	14.57–15.39	12.59–13.98	**0.00–1.00**	
4 *P. fraudulentella*	16.00–16.63	16.43–17.92	9.39–9.78	–

Genetic distances (%) were corrected with the Kimura two-parameter (K2P) substitution model using MEGA X; extreme values of intraspecific and interspecific distances are given (the numbers in bold are the intraspecific distances).

### Taxonomic account

#### Pelecystola

Taxon classificationAnimaliaLepidopteraTineidae

Meyrick, 1920 (New record for China)

CB3240B3-A48F-5862-84A5-61CD18F364B1

Pelecystola Meyrick, 1920: 103. Type species: Pelecystola decorata Meyrick, 1920, by original designation.
Zularcha Meyrick, 1937: 75. Type species: Zularcha melanochares Meyrick, 1937, by monotypy.
Neurozestis Meyrick, 1938: 25. Type species: Neurozestis polysticha Meyrick, 1938, by monotypy.

#### Pelecystola peculiaris sp. nov.

Taxon classificationAnimaliaLepidopteraTineidae

D8668241-9AB9-5768-8917-1467FFFB4587

http://zoobank.org/373DE18D-5C47-4FB6-BE4E-7B23DE670572

**Figures 2–5.**
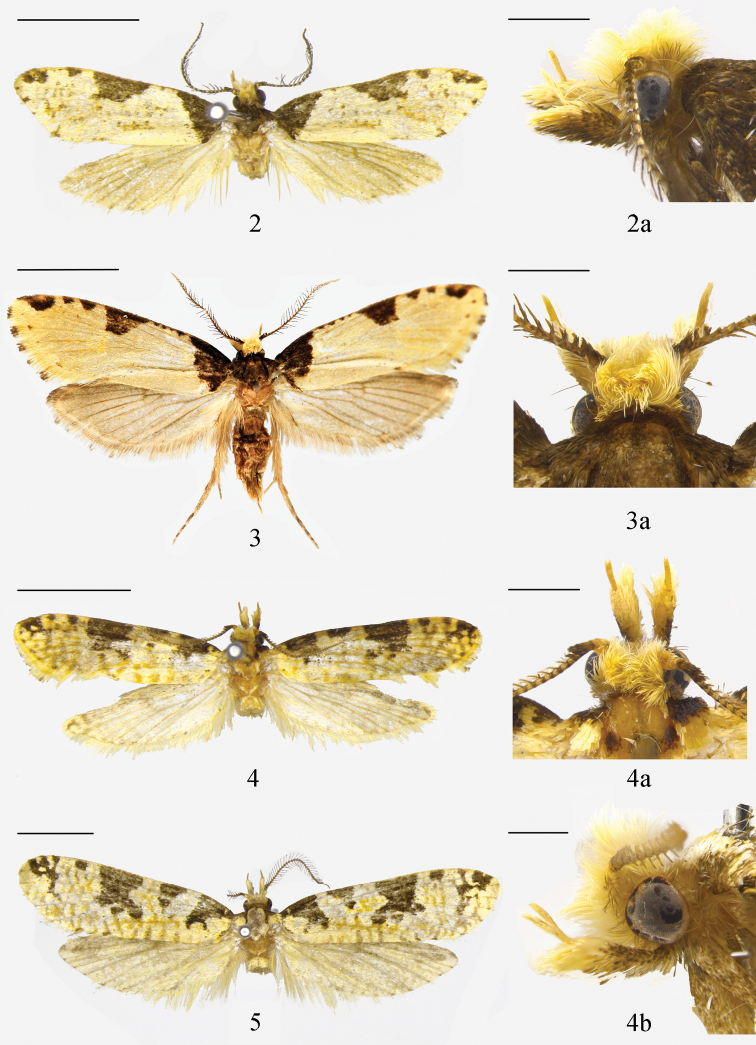
Adults **2, 3***Pelecystola peculiaris* sp. nov. **2** adult, holotype, male **2a** lateral view of head, paratype, male **3** adult, paratype, female **3a** dorsal view of head, paratype, female **4, 5***P. strigosa***4** adult, male **4a** dorsal view of head, male **4b** lateral view of head, male **5** adult, female. Scale bars: 5.0 mm (adults); 1.0 mm (heads).

**Figures 6, 7.**
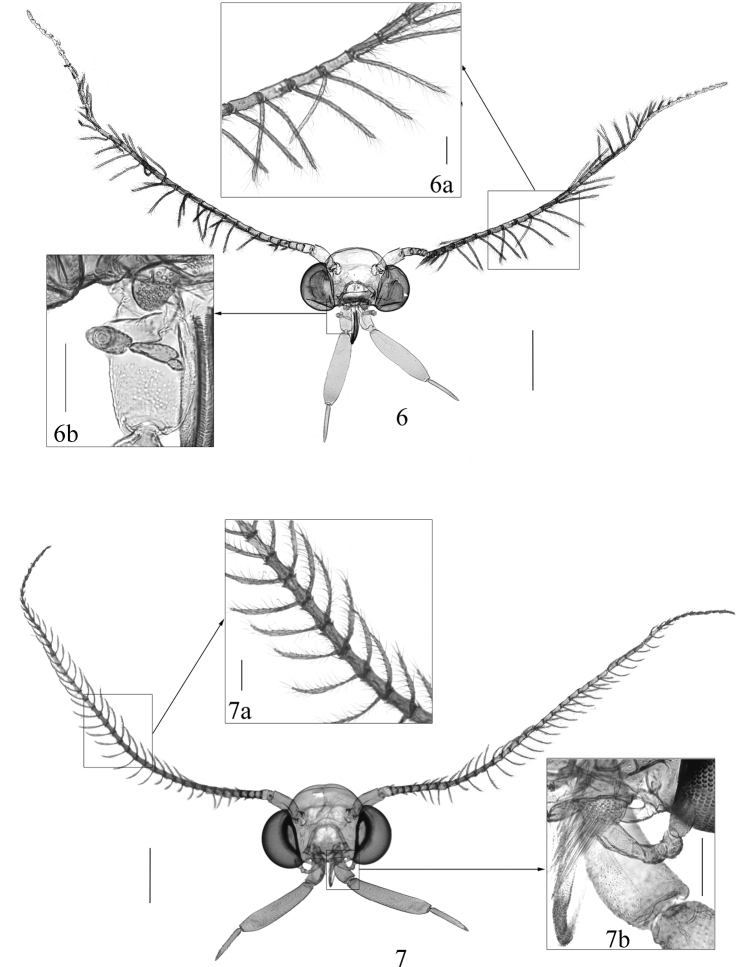
Head **6***Pelecystola peculiaris* sp. nov. **6a** flagellum **6b** Maxillary palpus, paratype, male, slide No. YLL20001 **7***P. strigosa***7a** flagellum **7b** maxillary palpus, male, slide No. YLL18066. Scale bars: 0.5 mm (**6, 7**), 0.1 mm (**6a, 6b, 7a, 7b**).

**Figures 8, 9.**
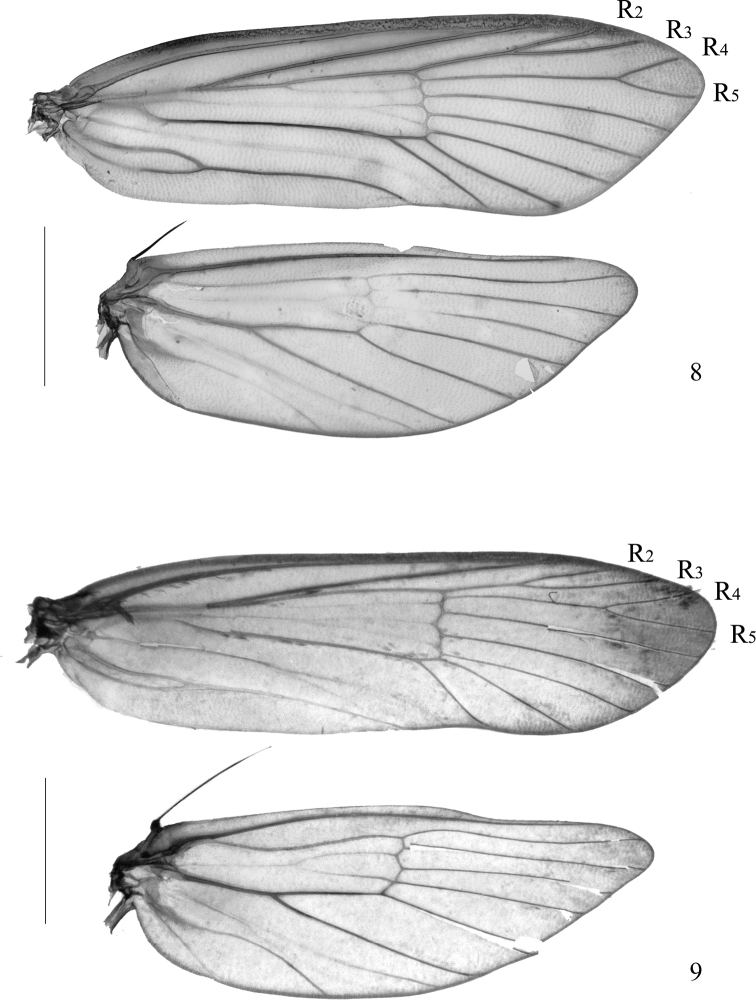
Venation **8***Pelecystola peculiaris* sp. nov., paratype, male, slide No. NKYLL022 **9***P. strigosa*, male, slide No. YLL18066. Scale bars: 2.0 mm.

**Figures 10, 11.**
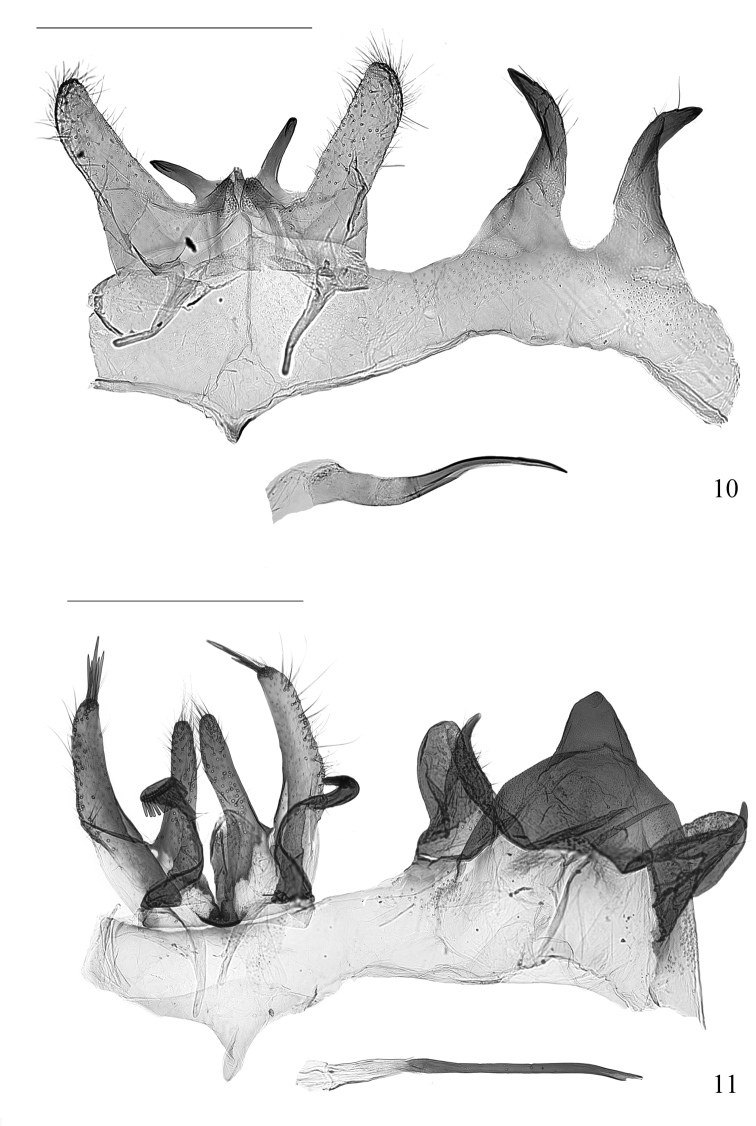
Male genitalia **10***Pelecystola peculiaris* sp. nov., holotype, slide No. DNAYLL18052 **11***P. strigosa*, slide No. YLL18067. Scale bars: 0.5 mm.

**Figures 12, 13.**
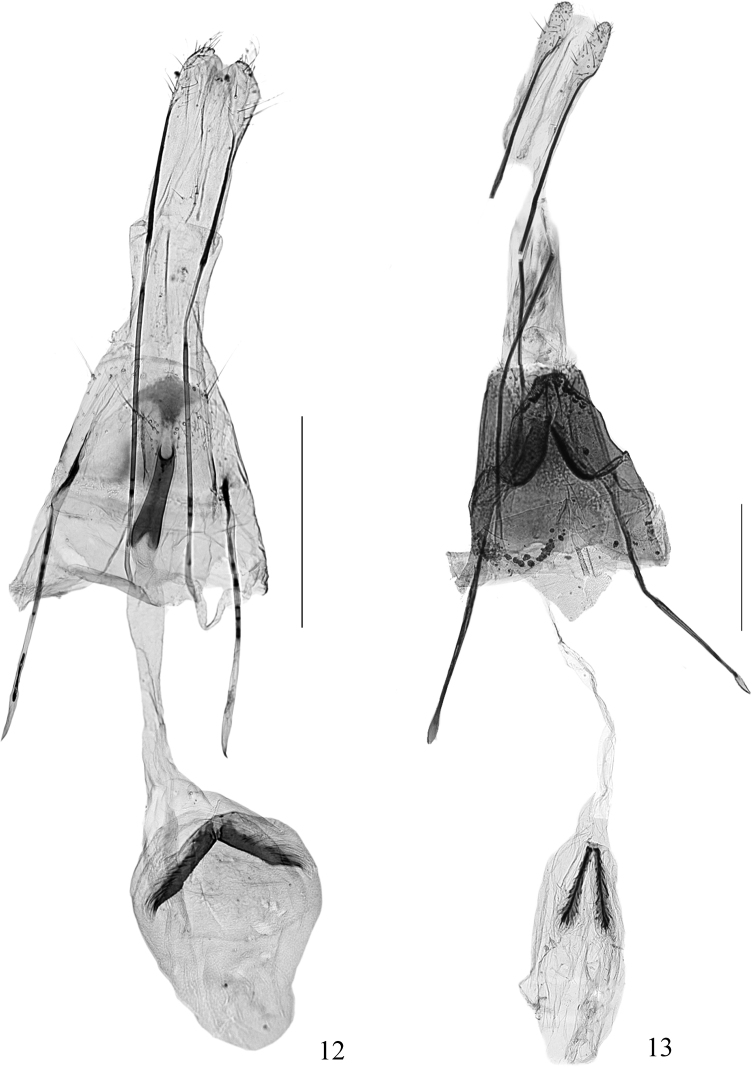
Female genitalia **12***Pelecystola peculiaris* sp. nov., paratype, slide No. DNAYLL18063 **13***P. strigosa*, slide No. DNAYLL18054 Scale bars: 0.5 mm.

Figures 2 , 3 , 6 , 8 , 10 , 12

##### Type material.

***Holotype*: China**: • ♂; Sichuan Province, Mianyang, Pingwu, Wanglang (32°54'N, 104°09'E); alt. 2569 m; 23-vii-2017; leg. Mujie Qi and Xiaofei Yang; genitalia slide No. DNAYLL18052. ***Paratypes***: **China**: • 3♂, 2♀; Henan Province, Neixiang County, Baotianman (33°12'N, 111°53'E); alt. 1200 m; 23–31-v-2006; genitalia slide Nos. NKYLL022, YLL09016 • 1♂; Henan Province, Song County, Mt Baiyun (34°08'N, 112°05'E); alt. 1580 m; 24-v-2002; leg. Xinpu Wang • 1♀; Shaanxi Province, Foping County, Yuebaxiang (38°19'N, 108°00'E); 22-vii-1985; leg. Houhun Li; genitalia slide No. XYL03193 • 1♀; Shaanxi Province, Langao County, Qiancenghe (32°07'N, 108°48'E); alt. 1338 m; 10-viii-2016; leg. Weixing Feng and Wentao Shi; genitalia slide No. DNAYLL18051 • 1♀; Fujian Province, Mt Wuyi, Guadun (27°44'N, 117°38'E); alt. 1100 m; 23-v-2004; leg. Haili Yu; genitalia slide No. YLL09014 • 1♀; Hunan Province, Sangzhi (29°44'N, 110°03'E), Tianpingshan; 11–13-v-2007; leg. Liusheng Chen • 1♀, Huixiangping, Mt Fanjing (27°55'N, 108°41'E), Guizhou Province; alt. 1700 m; 1-vi-2002; leg. Xinpu Wang, genitalia slide No. XYL03192 • 1 ♀; Huguosi, Mt Fanjing, Guizhou Province; alt. 1300 m; 1-viii-2001; leg. Houhun Li and Xinpu Wang, genitalia slide No. XYL02032 • 1♀; Guizhou Province, Mt Leigong (26°22'N, 108°11'E), Xiannvtang; alt. 1535 m; 25.VII.2019; leg. Mengran Xing et al.; genitalia slide No. DNAYLL18063 • 1♀; Guizhou Province, Mt Fanjing, Jinding; alt. 1300 m; 1-vii-2001; leg. Houhun Li and Xinpu Wang; genitalia slide No. XYL02031 • 1♀; Gansu Province, Tianshui, Dangchuan (34°37'N, 105°42'E), Huamiao; alt. 1331 m; 29-vii-2006; leg. Xinpu Wang and Xiangfeng Shi; • 1♀; Sichuan Province, Yaan (30°30'N, 102°54'E), Baoxing, Fengtongzhai; alt. 1565 m; 3-viii-2016; leg. Tao Fei, genitalia slide No. DNAYLL18064.

##### Diagnosis.

The new species is similar to *Pelecystola strigosa* in its venation, forewing pattern, and paired plume-like signa in the female genitalia, but the new species can be easily distinguished from the latter by genital morphology: the subovate valva has an elongated rod-like basal process on ventral margin in the male genitalia, and the simple eighth sternite is not folded ventrad in the female genitalia. In *P. strigosa*, the divided valva has a pedunculate pectinifer arising from base on costal margin in the male genitalia; and the eighth sternite is strongly folded and forming a tapered plate ventral to ostium in the female genitalia.

##### Description.

**Adult** (Figs 2, 3): wingspan 20.0 mm in holotype, 16.0–22.5 mm in paratype males, 19.0–26.0 mm in paratype females. Vertex and frons (Figs 2a, 3a) creamy yellow. Antenna (Fig. 6a) bipectinate in both sexes, ca 0.5× length of forewing, with scape violet-black on dorsal surface, yellowish white on ventral surface, pecten rusty brown, more than 20 bristles; flagellum bipectinate, covered with narrow appressed grayish yellow scales on dorsal surface, without scales on ventral surface. Labial palpus (Fig. 6) 2.0× height of head, segment ratios 1:2.5:1.1; yellow, first palpomere and base of second palpomere dark brown on outer surface; with a few dark bristles arising laterally along second palpomere; third palpomere slender, without vom Rath’s organ. Maxillary palpus (Fig. 6b) with five palpomeres, segment ratios 2:1:1:1:0.2. Thorax and tegula blackish brown. Forewing venation (Fig. 8) complete, R_2_ and R_3_ stalked at one-half, R_4_ and R_5_ with a long stalk; ground color pale yellow, shining orangish yellow from lower angle of cell to anal angle; patterned with a few black patches along costal margin: a triangular basal blotch inwardly oblique from costal 1/5 to dorsal 1/7; a trapezoidal patch at middle, its posterior margin not reaching to cell; a small semicircular patch near apex; four dots between basal blotch and medial patch, another one or two spots between medial and dorsal patches; termen with obscure dots at end of veins; fringe yellow, individual scales dark-tipped. Hindwing venation complete and separated; grayish yellow, with a slight coppery sheen; fringe grayish white with a dark median band at basal 1/3. Fore and mid legs black, hind leg yellowish brown except for tarsomeres brown at dorsal base.

***Male genitalia*** (Fig. 10). Tegumen gently concave on anterior margin, completely fused with vinculum into a ring. Saccus ca 2/5 length of valva, triangular, narrowly rounded at tips. Uncus lobe strongly sclerotized, long and horn-like, curved ventrad. Valva deeply divided into two lobes: cucullar lobe with a triangular base and an elongated rod-like distal part; saccular lobe subovate, rounded apically. Aedeagus slender, curved, tubular, gradually narrowed toward sharp apex, deeply channeled from apex to middle dorsally; vesica without cornutus.

***Female genitalia*** (Fig. 12). Eighth tergite rectangular, posterior margin with sparse long setae, slightly concave at anterior middle; eighth sternite straight on anterior margin, gently convex and with sparse long setae on posterior margin. Anterior apophysis 0.6× length of posterior apophysis. Ostium at center of eighth sternite, small, rounded. Antrum 2/7 length of ductus bursae (including antrum), cylindrical, triangularly concave on anterior end. Ductus bursae slightly longer than corpus bursae, slender, thin walled. Corpus bursae elongated suboval; signa consisting of pair of plume-like arms partially fused posteriorly at posterior 1/3 of corpus bursae, each arm with a strongly sclerotized, carinate stem. Inception of ductus seminalis at anterior end of antrum.

##### Distribution.

China (Fujian, Gansu, Guangdong, Henan, Hunan, Shaanxi, Sichuan).

##### Etymology.

The specific name is derived from the Latin *peculiaris* (= peculiar), referring to the special bipectinate antenna in both sexes that is peculiar in Tineidae.

##### DNA barcode.

Four DNA barcodes from the holotype and three female paratypes were generated and deposited in GenBank and BOLD systems: MW396737/PELE004-20 (for holotype), MT749675/PELE001-20, MW396736/PELE003-20, MW396738/PELE005-20 (for paratypes). For more details see the Suppl. material 1: Table S1.

#### Pelecystola strigosa

Taxon classificationAnimaliaLepidopteraTineidae

(Moore, 1888) (New record for China)

B4A94357-6C55-5E25-8547-2F40E45D47FC

Figures 4 , 5 , 7 , 9 , 11 , 13

Euplocamus strigosa Moore, 1888: 281.
Euplocamus hierophanta Meyrick, 1916: 617; Moriuti 1982: 163.
Semioscopis maculella Matsumura, 1931: 1093.
Pelecystola strigosa : Robinson et al. 1994; Robinson and Tuck 1996: 15; Sakai 2013: 135.

##### Material examined.

**China**: • 1♂; Yunnan Province, Ruili (24°00'N, 97°50'E), Rare Botanical Garden; alt. 1000 m; 5-viii-2005; leg. Yingdang Ren; genitalia slide No. YLL11078 • 1♂; Yunnan Province, Xishuangbanna (22°10'N, 100°51'E), Yexianggu; alt. 762 m; 17-vii-2014; leg. Kaijian Teng et al.; genitalia slide No. DNAYLL18053 • 2♂; Yunnan Province, Baoshan City (25°24'N, 98°45'E), Gaoligongshan, Baihualing; alt. 1470 m; 30-vii-2013; leg. Linlin Yang; genitalia slide Nos. YLL18067, YLL18070 • 1♂; Yunnan Province, Baihualing, Hanlongzhai; alt. 1577 m; 5-viii-2015; leg. Kaili Liu and Jingxia Zhao; genitalia slide No. DNAYLL18068 • 1♂; Yunnan Province, Dehong, Ruili, Rare Botanical Garden; alt. 1166 m; 17-viii-2015; leg. Jingxia Zhao; genitalia slide No. DNAYLL18065 • 1♂; Yunnan Province, Wenshan (23°10'N, 104°48'E), Masupo, Xiajinchang; alt. 1470 m; 27-vii-2016; leg. Kaijian Teng; genitalia slide No. DNAYLL18066 • 1♂; Hainan Province, Mt Jianfeng (18°50'N, 108°43'E), Tianchi; alt. 810 m; 30-iii-2008, leg. Bingbing Hu and Haiyan Bai; genitalia slide No. YLL13099 • 1 ♀; Xizang Autonomous Region, Bomi (29°51'N, 95°46'E), Sangdeng; alt. 2695 m; 21-viii-2017; leg. Mujie Qi, Xiaofei Yang; genitalia slide No. DNAYLL18054.

##### Diagnosis.

Adults with wingspan 17.5–21.0 mm in male (Fig. 4), 32.5 mm in female (Fig. 5). *Pelecystola strigosa* is characterized by the bipectinate antenna (Figs 4a, b, 7a); the forewing (Fig. 9) with R_2_ and R_3_ stalked less than half of their length, R_4_ and R_5_ stalked in basal 3/5, patterned (Figs 4, 5) with a subtriangular basal patch in basal 1/5, with its posterior margin reaching to fold, a trapezoidal blotch at middle, and a small irregular patch near apex; the wide-deeply bilobed uncus with an ovate pouch at base of each lobe, the tegumen with a prominent triangular protrusion, the deeply divided valva with an elongate, slender stalk that enlarges apically and bearing a pectinifer consisting of minute spines in the male genitalia (Fig. 11); and the eighth sternite is strongly folded and forming a tapered plate ventral to the ostium, the elongate and slender ductus bursae, and the paired signa with each arm slender and plume-like in the female genitalia (Fig. 13).

##### Distribution.

China (Hainan, Yunnan, Xizang), India, Japan, Malaysia (Sabah), Indonesia (Sulawesi).

##### Remarks.

*Pelecystola strigosa* was originally described in *Euplocamus* and later assigned to *Pelecystola* (Robinson et al. 1994; Robinson and Tuck 1996; Sakai 2013), with which it shares the peculiar pedunculate pectinifer arising on a long stalk from the costal base of the valva. The species superficially resembles *Pelecystola decorata* except for having bipectinate antennae in both sexes. Forewing venation in *P. strigosa* is quite different from its congeners. R_2_ and R_3_ stalked, R_4_ and R_5_ long stalked in *P. strigosa*, whereas all branches of R are separate in other *Pelecystola* species. Additionally, the maxillary palpus (Fig. 7b) in *P. strigosa* has four palpomeres, with a ratio of 2:1:1:1. In its allies, such as *P. nearctica*, there are five palpomeres.

##### DNA barcode.

Five DNA barcodes from four males and one female were generated and deposited in GenBank and BOLD systems: MW396739/PELE006-20, MW396740/PELE007-20, MW396741/PELE008-20, MW396742/PELE009-20, MT749676/PELE002-20. For more details see the Suppl. material 1: Table S1.

## Discussion

Our discovery of the new species dates back to 2010. During the preparation of specimens of the subfamily Euplocaminae, the first author found a species possessing bipectinate antennae in the females, which has never been observed in females of Euplocaminae. After examining its venation and genitalia structures, we supposed it belonged to the genus *Pelecystola*, as it shared the following characters with species of *Pelecystola*: deeply bilobed uncus and valva in the male genitalia and the paired plume-like signa in the female genitalia. However, the valva of the new species lacked the pedunculate pectinifer which was considered as a distinctive feature of *Pelecystola*. It was hoped that DNA barcoding might provide better resolution of the generic affiliation of the new species.

In the last few years, we were able to obtain some fresh specimens for DNA extraction and sequencing. As the genus *Pelecystola* is morphologically close to Euplocaminae and Scardiinae, in the initial study, we tried to reconstruct a molecular phylogenetic tree based on COI sequences partly obtained from our study and partly from published data of the two subfamilies from GenBank. The previous result showed the new species to form an independent clade, which made it a new genus. However, this conclusion was doubted by other experts, as the validity of the new genus was not sufficiently supported. Dr Marko Mutanen pointed out the weaknesses in the analysis and provided new evidence that the new species was nested within *Pelecystola* when the tree was built from COI combined with additional sequence data of Tineidae. Following his suggestions, we added more COI sequences of Tineidae for analysis, only to find that the systematic position of the new species was unstable. It is inadequate to fully resolve the generic affiliation of the new species based on only one gene marker and very limited species sampling.

We extended our gene selection to include the CAD and wingless genes in addition to COI, because the two nuclear markers could provide useful taxonomic information within Tineidae in previous studies (Mutanen et al. 2010; Regier et al. 2015). The generic affiliation of *Pelecystola peculiaris* sp. nov. was robustly confirmed according to the topology of the maximum likelihood tree (Fig. 1) reconstructed using the concatenated data set of the three gene fragments. The subfamily affiliation of *Pelecystola* was not resolved in this study. While pioneers have made significant efforts to provide morphology-based hypotheses (Robinson 1988; Robinson and Nielsen 1993; Robinson and Tuck 1997; Davis and Robinson 1998) and molecular phylogenetic analyses (Mutanen et al. 2010; Regier et al. 2015) of relationships within Tineidae, more than 800 species in about 200 genera of Tineidae have been placed in the polyphyletic Myrmecozelinae or left unattributed to subfamily (Robinson 2009; Regier et al. 2015). There is a remarkable lack of detailed morphological information in most of these species, which makes their identification rather difficult. In addition, specimens in collections of many species are few and old, and that significantly makes obtaining molecular samples from them difficult. These problems undoubtedly have impeded a comprehensive phylogenetic analysis with combined morphological and molecular evidence.

## Supplementary Material

XML Treatment for Pelecystola

XML Treatment for Pelecystola peculiaris

XML Treatment for Pelecystola strigosa

## Supplementary materials

Supplementary material 1 Table S1. Specimen data and GenBank accessions Data type species data https://binary.pensoft.net/file/558351This dataset is made available under the Open Database License (http://opendatacommons.org/licenses/odbl/1.0/). The Open Database License (ODbL) is a license agreement intended to allow users to freely share, modify, and use this Dataset while maintaining this same freedom for others, provided that the original source and author(s) are credited.Lin-Lin Yang, Hou-Hun Li

Supplementary material 2 Table S2. Species used in the phylogenetic analysis, with GenBank and BOLD references Data type molecular data https://binary.pensoft.net/file/558352This dataset is made available under the Open Database License (http://opendatacommons.org/licenses/odbl/1.0/). The Open Database License (ODbL) is a license agreement intended to allow users to freely share, modify, and use this Dataset while maintaining this same freedom for others, provided that the original source and author(s) are credited.Lin-Lin Yang, Hou-Hun Li

Supplementary material 3 Table S3. List of forward (F) and reserve (R) primers with annealing temperature used in PCR amplification Data type molecular data https://binary.pensoft.net/file/558353This dataset is made available under the Open Database License (http://opendatacommons.org/licenses/odbl/1.0/). The Open Database License (ODbL) is a license agreement intended to allow users to freely share, modify, and use this Dataset while maintaining this same freedom for others, provided that the original source and author(s) are credited.Lin-Lin Yang, Hou-Hun Li

Supplementary material 4 Table S4. PCR reaction system Data type molecular data https://binary.pensoft.net/file/558354This dataset is made available under the Open Database License (http://opendatacommons.org/licenses/odbl/1.0/). The Open Database License (ODbL) is a license agreement intended to allow users to freely share, modify, and use this Dataset while maintaining this same freedom for others, provided that the original source and author(s) are credited.Lin-Lin Yang, Hou-Hun Li

Supplementary material 5 Table S5. PCR thermal cycling Data type molecular data https://binary.pensoft.net/file/558355This dataset is made available under the Open Database License (http://opendatacommons.org/licenses/odbl/1.0/). The Open Database License (ODbL) is a license agreement intended to allow users to freely share, modify, and use this Dataset while maintaining this same freedom for others, provided that the original source and author(s) are credited.Lin-Lin Yang, Hou-Hun Li
